# New evidence on the earliest domesticated animals and possible small-scale husbandry in Atlantic NW Europe

**DOI:** 10.1038/s41598-020-77002-4

**Published:** 2020-11-18

**Authors:** Philippe Crombé, Kim Aluwé, Mathieu Boudin, Christophe Snoeck, Liesbeth Messiaen, Dimitri Teetaert

**Affiliations:** 1 Department of Archaeology, Ghent University, Sint-Pietersnieuwstraat 35, 9000 Ghent, Belgium; 2 Gate bv, Dorpsstraat 73, 8450 Bredene, Belgium; 3 Royal Institute for Cultural Heritage, Jubelpark 1, 1000 Brussels, Belgium; 4 Research Unit: Analytical, Environmental & Geo-Chemistry, Dept. of Chemistry, Vrije Universiteit Brussel, AMGC-WE-VUB, Pleinlaan 2, 1050 Brussels, Belgium; 5 G-Time Laboratory, Université Libre de Bruxelles, ULB, CP 160/02, 50, Avenue F.D. Roosevelt, 1050 Brussels, Belgium; 6 Maritime Cultures Research Institute, Dept. of Art Sciences & Archaeology, Vrije Universiteit Brussel, MARI-LW-VUB, Pleinlaan 2, 1050 Brussels, Belgium

**Keywords:** Stable isotope analysis, Archaeology, Palaeontology

## Abstract

The distribution of the first domesticated animals and crops along the coastal area of Atlantic NW Europe, which triggered the transition from a hunter-gatherer-fisher to a farmer-herder economy, has been debated for many decades among archaeologists. While some advocate a gradual transition in which indigenous hunter-gatherers from the very beginning of the 5th millennium cal BC progressively adopted Neolithic commodities, others are more in favor of a rapid transition near the end of the 5th millennium caused by a further northwest migration of farmers-herders colonizing the lowlands. Here, radiocarbon dated bones from sheep/goat and possibly also cattle are presented which provide the first hard evidence of an early introduction of domesticated animals within a hunter-gatherer context in NW Belgium, situated ca. 80 km north of the agro-pastoral frontier. Based on their isotope signal it is suggested that these first domesticates were probably not merely obtained through exchange with contemporaneous farmers but were kept locally, providing evidence of small-scale local stockbreeding in the lowlands maybe as early as ca. 4800/4600 cal BC. If confirmed by future in-depth isotope analyses, the latter testifies of intense contact and transmission of knowledge in this early contact period, which is also visible in the material culture, such as the lithic and pottery technology. It also implies direct and prolonged involvement of farmer-herders, either through visiting specialists or intermarriage, which follows recent genetic evidence demonstrating much more hunter-gatherer ancestry in early farmer’s genes in western Europe compared to central and SE Europe.

## Introduction

**Figure 1:**
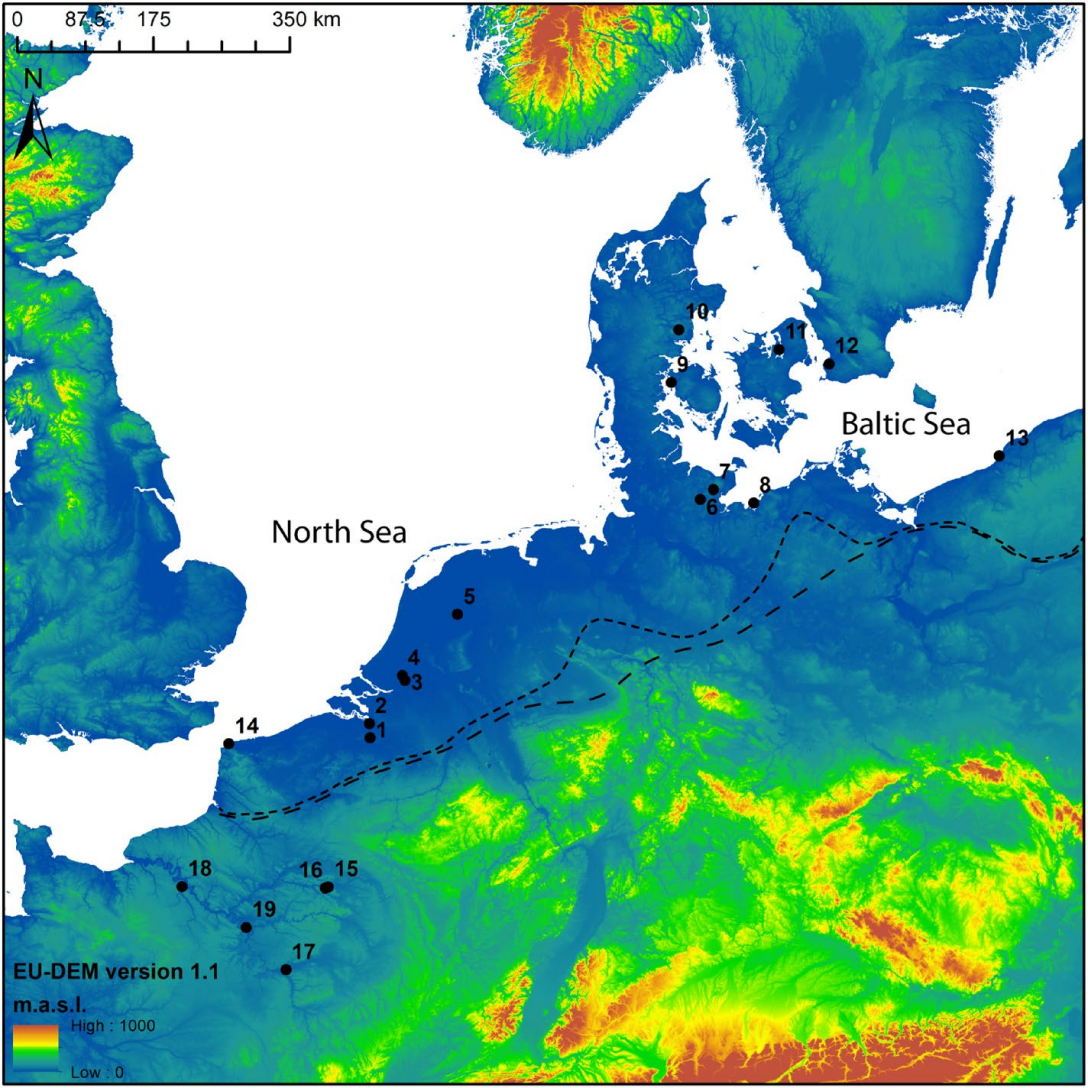
Elevation map of NW Europe (European Union, Copernicus Land Monitoring Service 2019, European Environment Agency (EEA)), indicating the agropastoral frontier between ca. 5300 and 4600 cal BC (wide dotted line) and between ca. 4600 and 4000 cal BC (narrow dotted line), as well as the contemporaneous sites discussed in this paper: 1. Bazel 2. Doel 3. Hardinxveld 4. Brandwijk 5. Schokland 6. Schlamersdorf 7. Rosenhof 8. Poel 9. Tybrind Vig 10. Ringkloster 11. Lollikhuse 12. Hindbygården 13. Dąbki 14. Mont d'Hubert 15.Cuiry-les-Chaudardes 16. Maizy 17. Balloy 18. La Villette 19. Bercy (F) (modified after ^12^).

The coastal lowlands of Northwest Europe are situated at the periphery of the extensive loess belt of Central and West Europe, which was colonized in the course of the 6th millennium cal BC by migrating farmers from the Near East and Anatolia^1–4^. The transition from (Mesolithic) hunter-gatherers to (Neolithic) farmers-herders in this coastal lowland area (Fig. 1) has been debated by numerous scholars over the past decades. The debate centers around two opposing models. The first advocates a long-term and gradual transition towards farming and herding^5–9^ starting early in the 5th millennium cal BC, a process in which local hunter-gatherers played a significant role (acculturation/cultural diffusion model). The second model, on the other hand, considers a rapid introduction of domesticates near the end of the 5th millennium cal BC, more precisely around ca. 4300 cal BC in the Netherlands^10,11^ and ca. 4100/4000 cal BC in the UK^12^, northern Germany^13,14^ and southern Scandinavia^15–18^. According to some, the speed of transition suggests demic diffusion of pioneering farmers from Central Europe into the lowlands of NW Europe^17,19^, as recently supported by genetic evidence^4^, although not all scholars agree on this^13,15,20^.

Central to this debate is the reliability and meaning of isolated finds of domesticated animal bones and cereal grains, claimed to pre-date 4300/4000 cal BC. The first model is strongly based on such finds, amongst which a few cattle and pig bones from the German Ertebølle site of Rosenhof LA 58 and 83^13^ and remains of cattle, sheep/goats and pigs from the Dutch Swifterbant sites of Hardinxveld “De Bruin”, Brandwijk and Schokland^8,9^, interpreted as proof of small-scale introduction of domesticates from at least 4700–4450 cal BC onwards (for a complete list of finds see^12^). However, most of these finds have been strongly questioned by the adherents of the second model using genetic^21^, stratigraphic and/or dating evidence^10–12,14,17^. Nonetheless, there are a few confirmed finds, such as pig remains with clear genetic connections to the Near East from the Ertebølle sites of Rosenhof and Poel dated 4720–4582 cal BC^22^ as well as a sheep/goat bone from Hardinxveld recently radiocarbon dated to 4520–4356 cal BC^11^. Yet, there is still debate about the nature and meaning of these first domesticates. At Rosenhof, for example, some scholars^23^ have argued that these early pigs might actually be hybrids between escaped (Neolithic) pigs and wild boar accidentally shot by hunter-gatherers, while at Hardinxveld it is suggested that early sheep/goat were probably animals on the hoof which might have been exchanged as gifts with farmers from the loess area. So currently there seems to be little substantial evidence in support of local husbandry within the NW European lowlands prior to 4300/4000 cal BC.

New dating and isotope evidence from the lowland of NW Belgium now lends support to the early presence of domesticates and possibly also small-scale husbandry within a hunter-gatherer context. The wetland site of Bazel “Sluis” yielded numerous carbonized cereal grains, mainly bread wheat (*Triticum aestivum* s.l.*/turgidum* s.l.). The oldest is firmly radiocarbon dated to ca. 4800 cal BC^24^. The same site also yielded animal bones, both wild and domesticated, the latter mainly belonging to cattle (*Bos primigenius f. taurus*) and to a lesser degree to sheep/goat (*Ovis ammon f. aries*)^25^. The presence of pig on the other hand remains difficult to assess due to known problems in differentiating between wild and domesticated specimens. Some initial radiocarbon dates^25^ indicated that these domesticated animals date back to ca. 4300 cal BC at the earliest, aligning the Scheldt basin with the chronology of the Netherlands^11^. However, further dating on newly discovered bones from the same site provided much older results as well as isotope information, which will be discussed here in the context of the Neolithization of the NW European lowland plain.

The site of Bazel “Sluis”, excavated in 2011, is situated in the floodplain of the river Scheldt, more precisely on a sandy elevation along a palaeochannel of the Scheldt. Thanks to the subsequent covering with peat(y) and alluvial clay sediments, the archaeological site is exceptionally well-preserved and is one of a few prehistoric sites yielding unburnt animal bones within the Scheldt basin. Based on diagnostic lithic^26^ and ceramic finds^27^ as well as extensive radiocarbon dating^28^, its use has been reconstructed in great detail, demonstrating a very long, albeit discontinuous occupation of the site. The site has known two major occupation phases, one dated to the 8th millennium cal BC corresponding to the Early and beginning of the Middle Mesolithic, and a second covering the late 6th till the first half of the 4th millennium cal BC, largely confined to a Late Mesolithic phase, and the Final Mesolithic/Early Neolithic Swifterbant and Michelsberg Culture period. In between these two phases the site seems to have been largely unoccupied. Unfortunately, due to the absence of sedimentation during this long period of site use, the remains of the two occupation phases are intermixed, making it particularly difficult to separate the archaeological finds and attribute them to specific occupation events^27,29^. However, as no bone material from the Early/Middle Mesolithic seemed to have survived, intermixing only applies to the bones from the second occupation phase^25^.

## Results

### Identification of domesticated taxa

Portion of the bone material (n = 420) was already studied and published in 2016^25^. Further analysis of the site revealed additional material (n = 995), without profoundly changing the overall species composition of the 2016-study (cf. SI 1). The majority of the previously unstudied assemblage cannot be identified to species level due to the more fragmented nature of this assemblage. The portion of completely unidentifiable fragments increased from 32 to 39%. However, when the remains were assigned to size-classes, it led to an increase of bone fragments from primarily medium-sized mammals, particularly, but also large mammals.

**Figure 2:**
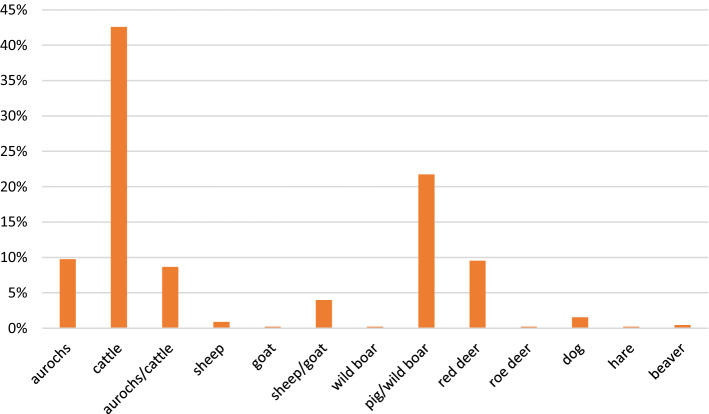
Species composition expressed in Number of Identified Specimens (NISP).

The assemblage consists of both wild and domesticated species (Fig. 2). The identified domesticated species include cattle (*Bos primigenius f. taurus*), pig (*Sus scrofa f. domestica*), sheep (*Ovis ammon f. aries*), goat (*Capra aegagrus f. hircus*) and dog (*Canis lupus f. familiaris*). Among the wild species are aurochs (*Bos primigenius*), red deer (*Cervus elaphus*), roe deer (*Capreolus capreolus*), hare (*Lepus capensis*), beaver (*Castor fiber*) and wild boar (*Sus scrofa*).

Cattle bones are predominant within the assemblage of identified mammal bones (42.6% of the total assemblage) and domesticated species. Although most parts of the skeleton are present, there seems to be an overrepresentation of horncore fragments and loose teeth (Table 1). Based on a small portion of bones and teeth that could give an indication of age of death, adult animals dominate the assemblage. Because of the fragmentation, only few measurements could be carried out (cf. Table 2).

**Table 1:** Skeleton parts of domesticated species, expressed in Number of Identified Specimens (NISP).

	Cattle	Sheep/goat
Horncore	60	2
Cranium	29	3
Maxilla	1	1
Mandible	3	
Teeth	44	5
Hyoid		1
Rib	7	1
Vertebrae	10	6
Scapula	3	
Humerus	4	
Radius	1	
Ulna	1	
Metacarpal	3	
Carpal	4	
Pelvis	2	2
Femur	3	
Tibia	5	1
Metatarsal	4	
Tarsal	1	
Astragalus	2	
Calcaneum	1	1
Metapodium	3	
Phalanx 1	1

**Table 2:** List of radiocarbon dated bone and teeth fragments of domesticated species and stable carbon and nitrogen data measured on collagen.

Sample code	Species	Element	Portion	Age	Measurements (mm)	Lab code ^14^C	Date BP	Calibrated age (2sigma)	%C	%N	δ^13^C	δ^15^N	C/N	Reference
2/22/10/4	*Bos primigenius f. taurus*	metacarpal	prox. < 1/2	prox	Bp = 59.46	RICH-26274	5852 ± 30	4795 (91.2%)4654BC 4639 (4.2%) 4612BC	34.1	12.3	− 24.6	6.6	3.2	This paper
2/23/7/1	*Bos primigenius f. Taurus?*	horncore	fragment			RICH-26280	5845 ± 33	4793 (95.4%) 4612BC	16.0	5.6	− 24.0	5.1	3.3	This paper
2/22/13/2	*Bos primigenius f. taurus?*	calcaneum	distal fragment			RICH-26281	5798 ± 30	4717 (95.4%) 4554BC	29.8	10.7	− 24.0	6.4	3.3	This paper
2/31/35/6	*Bos primigenius f. taurus*	metacarpal	prox. < 1/2	prox	Bp = 58.02	RICH-26275	5784 ± 29	4708 (95.4%) 4552BC	30.0	10.9	− 23.8	4.8	3.2	This paper
2/22/90/2	*Capra aegagrus f. hircus*	horncore	almost complete			RICH-26276	5753 ± 31	4692 (95.4%) 4520BC	18.7	6.3	− 23.1	6.0	3.5	This paper
2/22/32/1	*Ovis ammon f. aries/Capra aegagrus f. hircus*	tibia	fragment			RICH-26277	5729 ± 29	4683 (15.4%) 4631BC 4624 (80.0%) 4496BC	30.4	10.9	− 24.0	5.0	3.2	This paper
	*Ovis ammon f. aries*	cranium	fragment			KIA-47410	5320 ± 45	4318 (3.0%) 4296BC 4264 (91.5%) 4041BC 4013 (1.0%) 4004BC			− 23.5	5.3		Ervynck et al., 2016
	*Ovis ammon f. aries*	calcaneum				KIA-47425	5330 ± 45	4322 (5.2%) 4291BC 4266 (90.3%) 4043BC			− 24.2	5.2		Ervynck et al., 2016
2/22/98/2	*Bos primigenius f. taurus*	incisor	complete	no wear		RICH-27578	5313 ± 28	4235 (95.4%) 4049BC	20.6	7.3	− 23.2	6.0	3.3	This paper
2/22/95/1	*Bos primigenius f. taurus*	metatarsal	complete		GL = 220 Bp = 43.62 Bd = 51.99 SD = 23.48	RICH-26279	5289 ± 31	4233 (92.8%) 4040BC 4015 (2.7%) 4002BC	28.2	10.3	− 23.5	5.5	3.2	This paper
	*Bos primigenius f. taurus*	os centrotarsale				KIA-47397	5150 ± 40	4043 (71.8%) 3926BC 3877 (23.6%) 3805BC			− 23.3	5.4		Ervynck et al., 2016
2/22/24/2	*Bos primigenius f. taurus*	lower incisor	complete			RICH-27576	5119 ± 28	3978 (47.5%) 3913BC 3878 (47.9%) 3804BC	27.9	10.1	− 23	5.2	3.2	This paper
2/22/52/1	*Bos primigenius f. taurus*	upper P2	complete	slight wear	L = 16.75 B = 9.51	RICH-27577	5113 ± 28	3975 (42.7%) 3909BC 3879 (52.7%) 3802 BC	24.8	9.0	− 22.5	6.8	3.2	This paper
	*Bos primigenius f. taurus*	metatarsal				KIA-47413	5105 ± 40	3976 (95.4%) 3796 BC			− 23.3	5.3		Ervynck et al., 2016
2/23/14/1	*Bos primigenius f. taurus*	lower incisor	complete	slight wear		RICH-27575	5090 ± 28	3963 (35.1%) 3895BC 3881 (60.3%) 3800BC	21.6	7.7	− 22.7	6.5	3.3	This paper
2/24/44/1	*Bos primigenius f. taurus*	tibia	dist. < 1/2	dist. unfused		RICH-26282	4802 ± 28	3648 (20.6%) 3624 BC 3601 (74.9%) 3525BC	38.2	13.8	− 23.2	6.6	3.2	This paper

Remains of sheep and/or goat are less common (5.1% of the total assemblage). Few could be attributed either to sheep (two cranial fragments, a horncore fragment and a calcaneum) or goat (a horncore), with the majority being classified as belonging to sheep/goat. These include parts of the skull, spine and hind leg (Table 1). Despite the small sample size, different age groups seem to be represented: two fragments of an unfused pelvis belong to an animal younger than 11 months, two fragments of heavily worn deciduous fourth premolars could be assigned to animal(s) around 2 years old and fused vertebrae point to adult animals.

Among the bones classified as wild boar/pig (21.7% of the total assemblage), some will definitely belong to domesticated pig, although these cannot be selected due to identification problems.

### Radiocarbon dating

**Figure 3:**
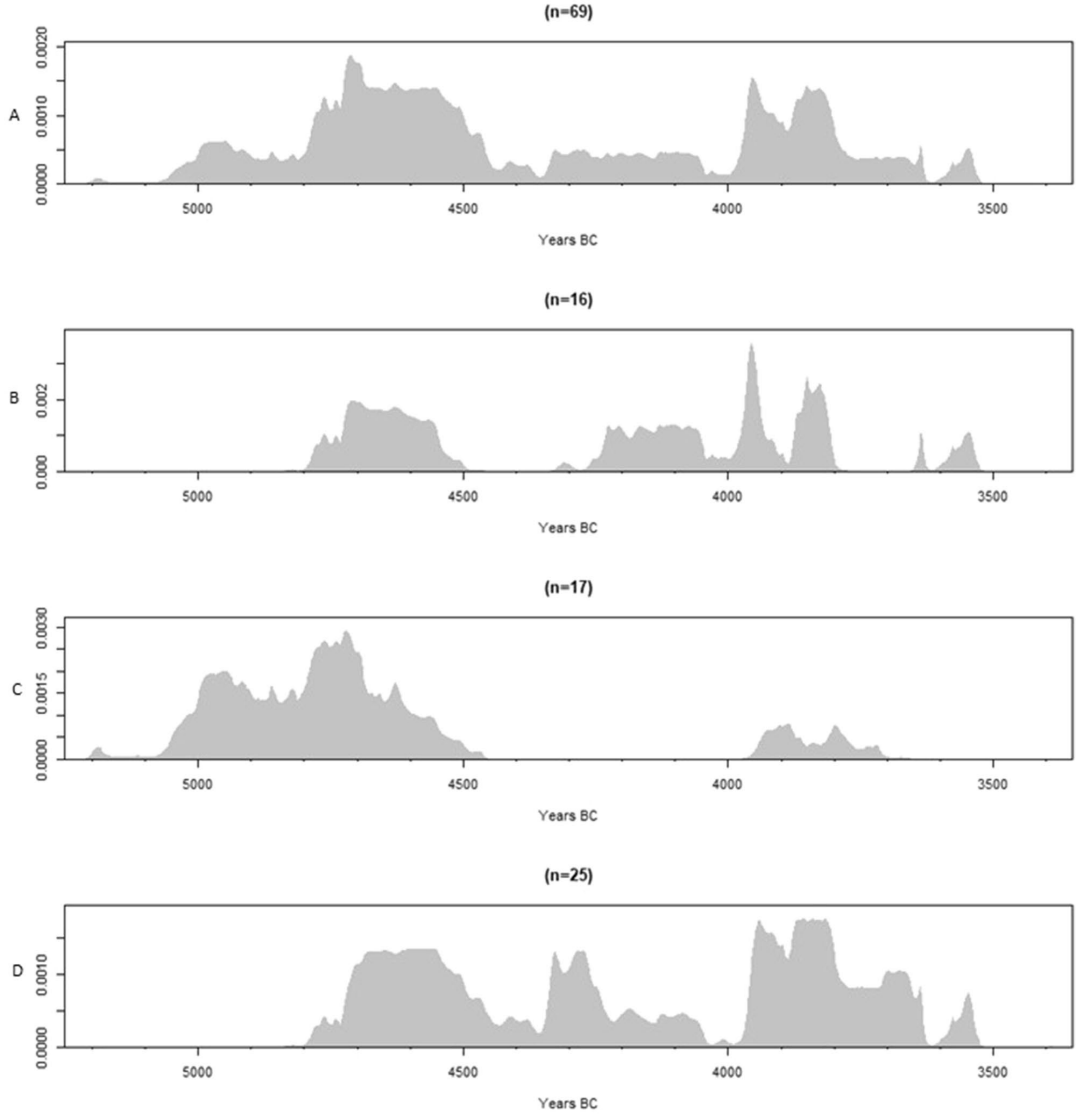
Sum probability distributions of calibrated radiocarbon dates: (**A**) All dated samples (carbonized hazelnut shells, charred cereals, bone, antler) representing the maximum duration of human occupation during the second phase; (**B**) Dates from domesticated herbivores (cattle, sheep/goat). (**C**) Dates from wild herbivores (aurochs, red deer). (**D**) Dates from charred cereals.

The radiocarbon dates obtained on a selection of cattle and sheep/goat remains (Table 2; Fig. 3B) clearly fall within two distinct clusters: one (n = 6) centered between ca. 4800 and 4500 cal BC and a second (n = 10) between ca. 4300 and 3800 cal BC, with an outlier around 3650–3525 cal BC. The total lack of dates in between these clusters, corresponding to the third quarter of the 5th millennium, most likely reflects a bias due to increased fluvial activity. This is likely to have caused erosion of the peat cover along the channel bank which included most of the bone assemblage^30^. A larger gap also starting around 4500 cal BC is observable in the radiocarbon evidence from the wild game found in the same stratigraphic context (Fig. 3C). However, this chronological gap is absent from the modelled dates on charred cereals (Fig. 3D), which were selected randomly over the entire site, and confirm that there has been no hiatus in the occupation of the site during the 5th millennium cal BC.

**Figure 4:**
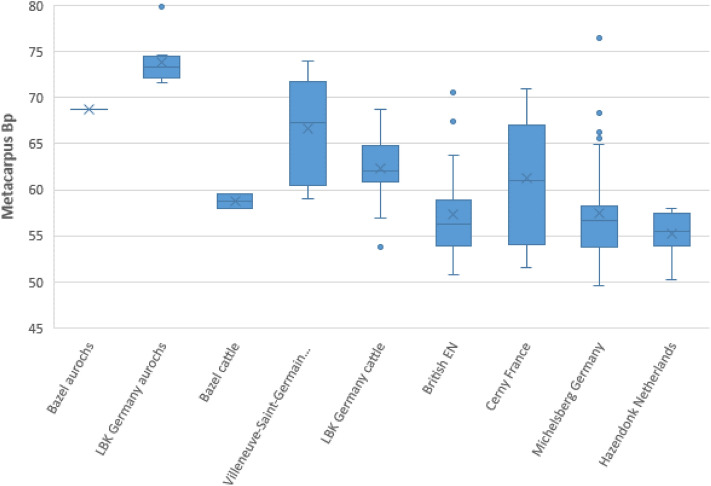
Size graph Bp metacarpal of cattle and aurochs at Bazel-Sluis and different Early and Middle Neolithic sites in France, Germany, Great-Britain and the Netherlands based on the EuroEvol data-set^65^.

Interestingly, the two radiocarbon clusters with domesticated animals include remains of both cattle and goat/sheep. Within the oldest cluster, there is an almost complete horncore from a goat and a cranium fragment from a goat or sheep. These two finds provide the strongest evidence of the early presence of domesticated animals within the Scheldt basin, as there is no doubt about their domesticated status. Wild ancestors of sheep and goat, resp. *Ovis orientalis* and *Capra aegagrus*, did not occur within Holocene Europe^31,32^, hence all sheep/goat bones retrieved from archaeological contexts belong to domesticated specimens. Similarly, a domesticated status is assumed for the four dated cattle bones from the oldest cluster. Two dates were performed on metacarpal bones which are proximally fused and, based on their appearance, do not belong to neonates. These bones have a proximal width of respectively 59 and 58 mm (Table 2), which perfectly matches that of domesticated specimens in several contemporaneous (Middle) Neolithic cultures, in particular the Cerny Culture (Fig. 4) and deviates considerably from aurochs. Based on this a domesticated status for these animals seems highly probable although it cannot be fully excluded that they represent very small female aurochs or that interbreeding between wild and domestic cattle took place^21^. One of these metacarpals provided the oldest radiocarbon date of the bone assemblage, situated between 4770 and 4690 cal BC (1 sigma) or 4795 and 4620 cal BC (2 sigma). The two remaining cattle bones from the oldest cluster unfortunately are too fragmented to yield reliable measurements, their attribution being based on overall dimensions of the fragments.

### Stable isotope data

**Figure 5:**
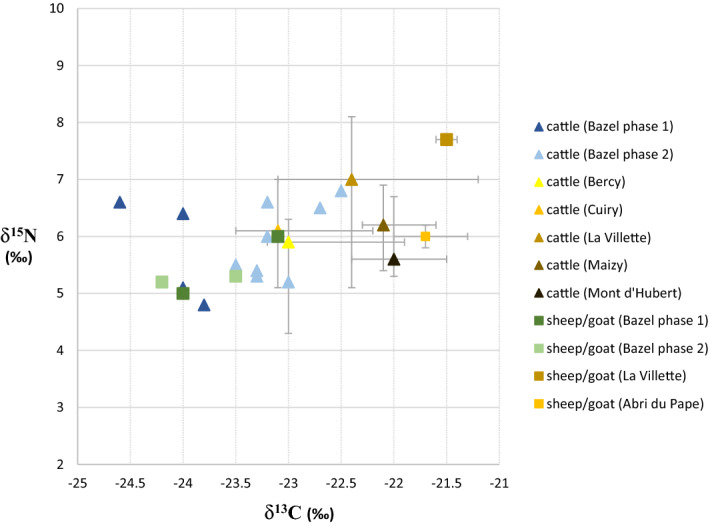
Carbon and nitrogen stable isotope data obtained on bone and dentine collagen of domesticated animals from the site of Bazel, compared to data from early and middle Neolithic sites in the adjacent loess region of France^33^.

The δ^13^C and δ^15^N values measured on bone collagen of all dated domesticated cattle and sheep/goat samples (Fig. 5; Table 2) form a very tight cluster, with δ^13^C values between − 24.6‰ and − 23.1‰ (mean 23.7‰) and δ^15^N values between 4.8‰ and 6.6‰ (mean 5.6‰). There are no extreme differences between samples of the oldest and youngest cluster, except a slight increase of the δ ^13^C values of 0.4‰ in average. The oldest samples tend to cluster around − 24‰, and the younger around − 23.5‰. The δ^13^C and δ^15^N values measured on cattle tooth dentine collagen (all belonging to the younger cluster) are slightly higher and range from − 23.2 to − 22.5‰ and 5.2 and 6.8‰ respectively.

The strontium isotope ratios (^87^Sr/^86^Sr) of the five analyzed cattle tooth enamel range from 0.7094 to 0.7121 and have concentrations going from 187 to 250 ppm (Table 3). The carbon and oxygen isotope ratios (δ^13^C_ap_ & δ^18^O_ap_) of these five teeth range from − 15.3 to − 12.8‰ and − 7.5 to − 4.6‰ respectively (Table 3). Only four of these teeth could be radiocarbon dated and all four belong to the younger cluster of radiocarbon dates. The δ^13^C_ap_ values and strontium concentration data group these teeth in two group: three teeth with higher δ^13^C_ap_ values (≥ − 13.5‰) and lower strontium concentrations ([Sr] < 200 ppm) and two teeth with lower δ^13^C_ap_ values (≤ − 14.5‰) and higher strontium concentrations ([Sr] > 240 ppm).

**Table 3:** Carbon, oxygen and strontium isotope results and strontium concentrations of cattle tooth enamel; *strontium concentration data normalized to calcium concentration of 40%.

Sample code	Species	Element	δ^13^C_ap_ (‰—VPDB)	δ^18^O_ap_ (‰—VPDB)	^87^Sr/^86^Sr	2σ	[Sr]* (ppm)
2/22/98/2	*Bos primigenius f. taurus*	lower incisor	− 12.8	− 6.9	0.710775	0.000010	188
2/22/24/2	*Bos primigenius f. taurus*	lower incisor	− 14.5	− 7.5	0.712147	0.000007	243
2/22/52/1	*Bos primigenius f. taurus*	upper P2	− 13.5	− 5.3	0.709906	0.000010	187
2/23/14/1	*Bos primigenius f. taurus*	lower incisor	− 15.3	− 7.1	0.709407	0.000012	250
2/22/52/1	*Bos primigenius f. taurus*	Upper P3	− 13.0	− 4.6	0.709701	0.000009	192

## Discussion

### Cultural assignment of the earliest domesticated animals

Based on the radiocarbon evidence from the wetland site of Bazel presented here, it can be concluded that domesticated animals, in particular sheep/goat and most likely also cattle, were already present in the lower Scheldt basin from the first half of the 5th millennium cal BC, more specifically from ca. 4800–4600 cal BC onwards. This confirms previous assumptions about early domesticates on the site, based on the presence of a possible, though not confirmed, mandible from a domesticated pig, dated 5830 ± 35 ^14^C BP or 4790–4590 cal BC (2 sigma)^25^. Furthermore, the timing of the earliest domesticated animals is perfectly synchronous with the first appearance of cereal grains on the site, mainly belonging to bread wheat^24^. All this implies that domesticates were introduced into the lowlands of Atlantic NW Europe, at least in the Lower Scheldt basin situated ca. 80 km from the agricultural border, earlier than previously assumed, which lends support to the long-term and gradual Neolithization model (cf. 1 Introduction).

However, given the long-term occupation of the site of Bazel (late 6th till mid-4th millennium cal BC) and the mixed contexts the cultural assignment of these earliest domesticates is not straightforward. As mentioned in the introduction, the studied bone fragments as well as cereal grains have been found intermixed with material remains, mainly lithic artefacts and pottery, belonging to various cultural traditions^26,27^, including both local, indigenous traditions (e.g. Late Mesolithic, Swifterbant and Michelsberg Cultures) and Neolithic Cultures known from the nearby loess region (e.g. late Linearbandkeramik (LBK) and epi-Rössen/Bischheim Cultures). However, the vast majority of these finds clearly belongs to indigenous traditions, hence it seems reasonable to assume that the domesticated animal bones as well as cereals can be attributed to these occupations. Among the ca. 8000 studied potsherds a maximum of 15% can be defined as “exotic” pottery on the basis of techno-morphological and decoration criteria, while the bulk has been locally produced using local clays. Furthermore, no exotic pottery contemporaneous with the oldest cluster of domesticated animals (ca. 4800 and 4500 cal BC) was found at the site, suggesting that the latter were most likely not imported by farmer-herders from the loess region, e.g. in the context of excursions and scouting expeditions in search of natural meadows (transhumance) and new arable lands and resources. This is corroborated by the lack of lithic settlement waste that can be linked to such occupations. Hence it is more likely that the earliest domesticated animals and cereals were obtained by local hunter-gatherers from the Lower Scheldt basin through contact with farmer-herders during their yearly migration into the nearby loess area^27^. Migration from the Lower Scheldt basin towards the loess region is well-established by the presence of high-quality lithic raw materials, mainly flint and Wommersom quartzite, originating from different regions within the Belgian loess area and used to produce “indigenous” tools. This said, it is not totally excluded that the first domesticated animals, or some of these, might be feral animals, which escaped from the loess area and were subsequently hunted by local hunter-gatherers. Though this seems rather unlikely given the fact that the early domesticates at Bazel include both sheep/goat, cattle and possibly also pig. Furthermore it does not explain the presence of cereal grains at the site, which appeared at the same time. The latter seems to be more in favour of the interaction and exchange theory. Either way the data from Bazel irrefutable proves that domesticated animals were present in the Lower Scheldt basin from 4800/4600 cal BC onwards and were thus within direct reach of the indigenous hunter-gatherers.

### Local husbandry versus (gift) exchange

The question that follows from this relates to the nature of these first domesticates in the Lower Scheldt basin. Earlier it has been suggested that it is very unlikely that the cereal grains dated prior to ca. 4000 cal BC indicate local agriculture, given the total lack of complementary evidence, such as cereal pollen and chaff, stone sickles and querns, and tillage marks^10,24^. The oldest cereals probably were part of a socio-economic exchange system that went beyond ‘acquiring the exotic’. The rather late transition to agriculture probably results from the unsuitability of the local wetland environment for soil cultivation. However, this does not necessary apply to the oldest domesticated animals, as husbandry is less dependent on local environmental conditions.

**Figure 6:**
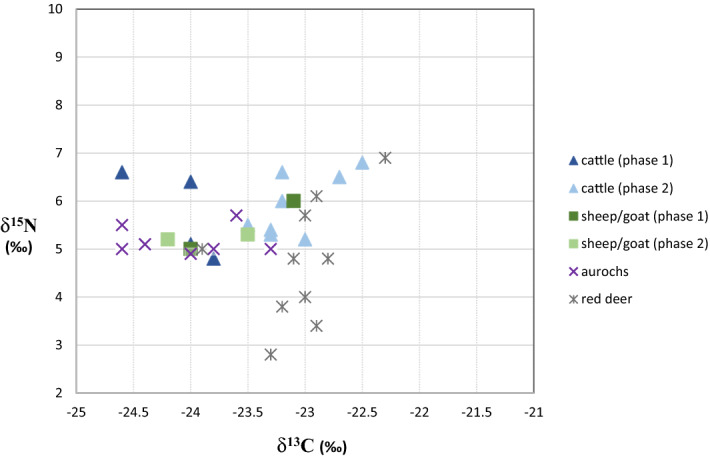
Carbon and nitrogen stable isotope data obtained on bone and dentine collagen of domesticated and wild herbivores^25^ from the site of Bazel.

Local husbandry might be indirectly deduced from the frequency of domesticated animals within the total bone assemblage. According to Zvelebil’s availability model^5^ the transition towards a Neolithic agropastoral economy, termed the substitution phase, is defined by the occurrence of between 5 and 50% of domesticates within the diet. Unfortunately, the ratio between wild and domesticated animal species cannot be determined at Bazel given the palimpsest situation. The only way to obtain that kind of information is by radiocarbon dating the entire bone assemblage, which is unfeasible. Nonetheless, the available dates on bones from wild game (n = 17; Fig. 3C) indicate that only few (n = 2) are contemporaneous with the youngest cluster of domesticated animals (Fig. 3B). This can be considered as a strong indication that, at least from ca. 4300 cal BC, local husbandry constituted the main part of the subsistence at the site. This is supported by the carbon and nitrogen isotope ratios measured on bone and dentine collagen (Table 2). Overall the stable carbon and nitrogen isotope values do not match well with those from cattle and goat remains collected on early (ca. 5300–4600 cal BC) and middle Neolithic sites (ca. 4600–3800/3700 cal BC) within the surrounding loess region of northern France and Germany, which generally yield somewhat higher δ^13^C and δ^15^N values^33–35^ (Fig. 5). On sites in northern France, situated closest to the site of Bazel (< 300 km), cattle have mean δ^13^C values of − 22.4 ± 0.6‰ (min. − 23.5‰, max.− 21.2‰) and mean δ^15^N values of 6.6 ± 0.8‰ (min. 5.1‰, max. 8.1‰). There is a relatively close match with the cattle bones from the LBK site of Cuiry-les-Chaudardes, however only for the youngest cluster from Bazel. For sheep, there is only data from two samples with a mean δ^13^C of –21.5 ± 0.1‰ and mean δ^15^N of 7.7 ± 0‰. Similarly high isotope values have been obtained on sheep/goat from sites in eastern France and western Germany^35^. These differences in isotope values might suggest that the first domesticated animals (or parts of these) in the Scheldt basin did not originate from or were not exchanged with the French nor German loess area. However, this does not directly imply that they were raised locally in the Scheldt valley, as they may have been obtained through contact with farming communities from the nearby (< 100/150 km) Belgian loess area from which unfortunately very little isotope data is currently available^36^(Fig. 5, site of Abri du Pape). However, as it has been demonstrated that stable carbon and nitrogen isotopes vary on a latitudinal scale across western Europe^33^ it can be expected that cattle and sheep/goat found closer to the Scheldt basin, i.e. north of 50°N, will have somewhat lower δ^13^C and δ^15^N values. On the other hand, the values for both domesticated species at Bazel correspond perfectly with those of wild herbivores (Fig. 6), in particular aurochs, present in the site and dating contemporaneously with the oldest cluster of domesticated animals (Fig. 3C). This could suggest that both domesticated and wild herbivores lived in similar environments and feeding habitats, as it has been amply demonstrated that δ^13^C signatures are closely linked to the natural environmental parameters of the local habitats^33^. Although this does not necessarily mean that both groups of herbivores lived in the same region, it might be an indication of small-scale local husbandry even from the very beginning.

This is also partly suggested by the strontium evidence. The strontium isotope ratios ranging from 0.7094 and 0.7121 are consistent with an origin in Belgium, at least for the domesticated cattle from the youngest cluster. However, the lack of adequate biologically available strontium baseline for Belgium makes it difficult at present to refine this interpretation. Such baselines exist for various countries^37–39^ and is currently being created for Belgium^40^. As such, this data should be revisited once this baseline becomes available. While it is not possible to exclude a Belgian origin or be more precise about the possible region(s) of origin within Belgium, the large spread in the strontium isotope data clearly shows that the animals were not living/grazing in the same areas. This is further supported by the strontium concentrations and carbon isotope ratios measured on the tooth enamel of these cattle (Table 3). Indeed, even if the sample size is extremely small (only five teeth), the fact that they cluster in two groups further suggest they were not consuming the same food. The oxygen isotope results also suggest these cattle did not grow/graze in the same place as their bulk oxygen isotope ratios range from − 7.5 to − 4.6‰. Two of the five specimen have oxygen isotope ratios (− 5.3 and − 4.6‰) consistent with the results obtained on modern horse teeth from the Scheldt basin (very close to Bazel) that exhibit seasonal variation between − 7 and 0‰ with an average value around − 4.5‰^41^. The other three samples with δ^18^O_ap_ values between − 7.5 and − 6.9‰ are similar to values seen in Roman and Medieval sheep from salt-marshes and salt-meadows in the nearby Belgian coast with average values between − 8.3 and − 6.3‰^42^. The limited amount of comparative data and the low number of sample analyzed here make it difficult to fully explain this variability. Either these cattle originate from different areas, possibly within Belgium but other places are also possible, or they were all grown locally but fed in different environments. The latter is a very plausible explanation given the environmental diversity around the site of Bazel especially during the second half of the 5th millennium cal BC, with dry coversands, mudflats, saltmarshes and peaty marshlands within a radius of 10 km^28^ (see below). However, more specimen from the Belgian Neolithic should be analyzed to provide a clearer picture of what was going on in terms of husbandry in Bazel.

### Shifting isotope ratios

In this context, the observed weak increase in the δ^13^C value between domesticates from the oldest to the youngest cluster might be of some interest. Various factors may have caused this increase, such as changes in precipitation, forest cover, and/or animal food. Recent research^43^ has demonstrated that cattle and sheep/goat which were seasonally fed with leaf fodder collected in the forest understory tend to display lower δ^13^C values, compared to animals grazing in open pastures. This is the result of the “canopy-effect”, caused by the accumulation of ^13^C-depleted CO_2_ in understory plants growing in dense and dark forests^44^. At two contemporaneous sites nearby Bazel – the sites of Doel-Deurganckdok sector B and M – exceptionally high amounts of charred ivy (*Hedera helix*) seeds and mistletoe (*Viscum album*) charcoal have been interpreted as possible remains of winter fodder used during winter/early spring ^(45,46 )^. Both evergreens are known to have been used as fodder widespread over Europe from the Middle Neolithic until Medieval times to compensate for restricted availability of grass during dry summers or snowy winters and/or over periods of stalling (^45^ *and references therein)*^.^. Both sites at Doel have been securely dated to the second half of the 5th millennium cal BC^28^, thus falling chronologically in between the two clusters of domesticated animals at Bazel and partially overlapping with the youngest cluster. Alternatively, the increase in δ^13^C towards the end of the 5th millennium may reflect changing environment. Regional pollen and macro-remains^45–47^ point to an extensive marshy environment characterized by a dense alder carr vegetation during the first half of the 5th millennium and thus contemporaneous with the oldest cluster of domesticated animals at Bazel. From ca. 4600 cal BC onwards the environment drastically changed into a brackish to freshwater tidal area under direct influence of sea level rise in the adjacent North Sea basin^47,48^. In the higher intertidal zone bordering the tidal marshes and creeks, an alluvial softwood forest dominated by willow and reed vegetation appeared, while on the higher grounds an alluvial hardwood forest was installed, a type of forest consisting of oak, elm, ash and a rich variety of shrubs and climbers, that was only rarely flooded. This more open environment probably provided animal fodder that was less ^13^C-depleted resulting into higher δ^13^C values in the animal bones.

In conclusion, stable carbon and nitrogen isotope ratios measured on collagen as well as plant macro-remains tend to support local husbandry rather than the importation of sheep/goat and presumably also cattle from farmer communities in the southern loess area, at least from the middle of the 5th millennium onwards, and possibly even earlier. The oxygen and strontium isotope results, however, suggest that the cattle, at least during the late 5th millennium cal BC, grew and/or grazed in different environments, which might have been locally available, albeit it cannot be fully excluded that some were obtained from further afield.

### The Neolithization process of the Lower Scheldt basin

**Figure 7:**
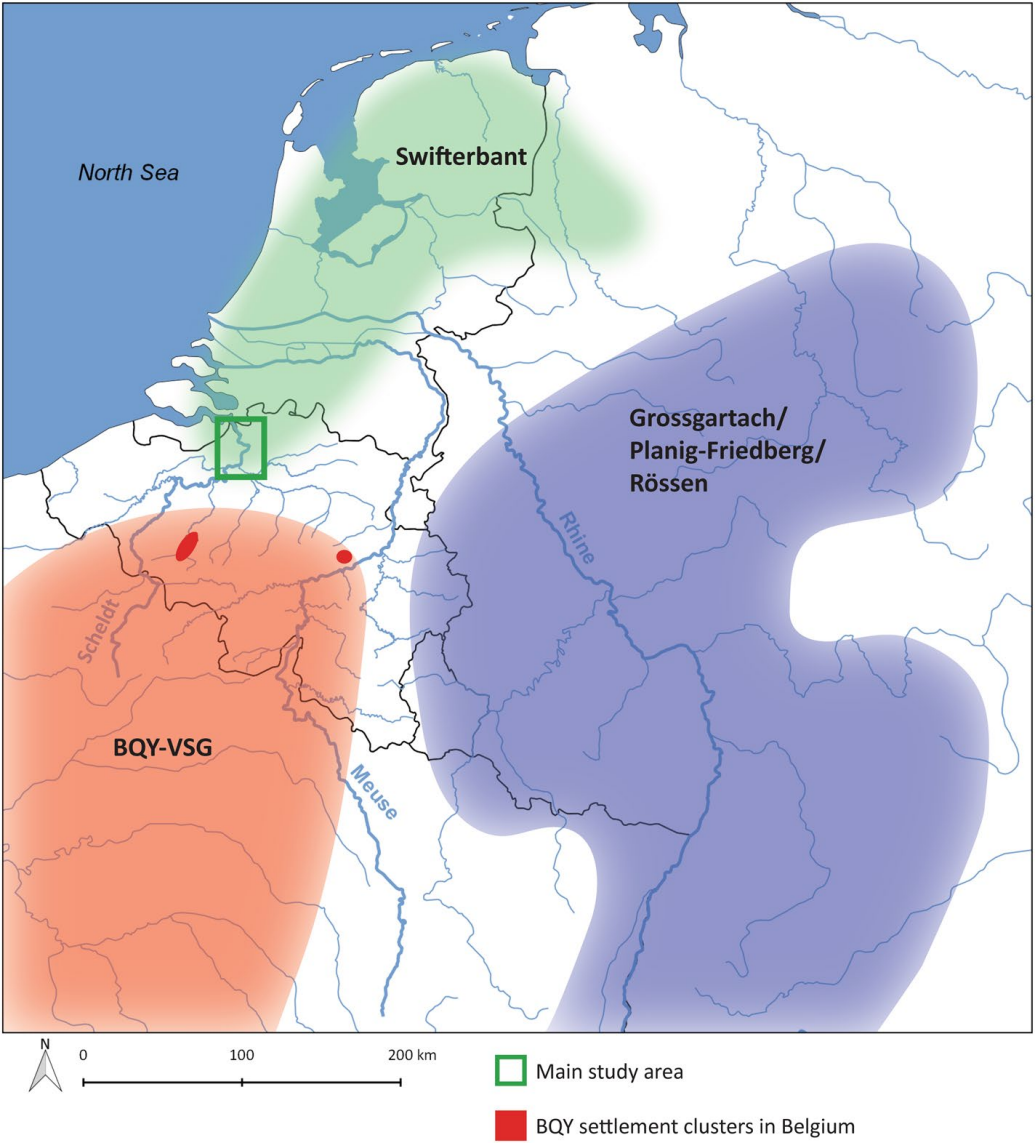
Main distribution area of the Swifterbant Culture, Blicquy/Villeneuve-Saint-Germain Culture (BQY/VSG) and Grossgartach/Planig-Friedberg/Rössen Cultures. For the latter, the map shows the extension of the Rössen Culture, which encompasses the earlier distribution areas of the Hinkelstein, Grossgartach and Planig-Friedberg. Green square = Lower Scheldt valley (Map designed using QGIS software version 2.18.17.https://qgis.org/downloads/).

The presence of domesticated animals and possible small-scale husbandry from ca. 4800–4600 cal BC onwards, implies that farmer/hunter-gatherer interaction along the NW border of the agro-pastoral frontier was much more intense and drastic than previously thought, and may have involved more than mere exchange of “exotic” goods. Indeed, local stockbreeding demands a transmission of knowledge e.g. through training by skilled specialists which implies direct and prolonged involvement of farmers/herders from the loess areas. This is corroborated by the material culture, which also underwent drastic changes at precisely the same moment. Between 4800 and 4600 cal BC new knapping techniques appeared within the local “Mesolithic” lithic traditions, focusing on the production of thick flakes and new tool types, such as splintered and facetted tools. This was accompanied by new activities, as indicated by new types of micro- and macroscopic usewear traces on lithic tools, e.g. crushing and grinding of animal bone^49^. The start of local pottery production by hunter-gatherers of the Scheldt river valley (Swifterbant Culture), using local clays, must have started during this same period^50^. These new technologies present clear affinities with post-LBK cultures, in particular with the Blicquy/Villeneuve-Saint-Germain Culture (ca. 5000/4950 – 4750/4650 cal BC) and later the Rössen Culture (ca. 4700 – 4450 cal BC) (Fig. 7). As such, these changes point to increased influence of farmer-herders from the loess area. The transfer of technological know-how must have required close and long-term interactions between the farmer-herders and hunter-gatherer population groups, which might have involved the migration of specialists or even intermarriage. This is certainly the case for the pottery production, which represents an entirely new technology in hunter-gatherer context, that required new knowledge of raw materials and the development of new motor habits, i.e. motions and body postures that were previously not used for other activities^51,52^. The same holds for keeping local livestock consisting of different species.

At first glance, all this seems in contradiction with genetic evidence from the Central European loess area, mainly from central and southern Germany^1–4^, which points to limited (< 10%, max. 17%) interaction and admixture between both populations before ca. 4000 cal BC, and thus genetic continuity over a period of ca. 1500 years. However, the first recently published genetic evidence from northern France^53,54^, demonstrates much more hunter-gatherer ancestry in early farmer’s genes in western Europe compared to central and SE Europe, which fits the conclusions of the present study. Interestingly, the predominance of cattle over sheep/goat within the assemblage of Bazel perfectly mirrors the livestock within the different succeeding agro-pastoral cultures of the loess region, characterized by a cattle-based economy in which sheep/goat only played a minor role (ca. 6/10% to 20%)^55^. This cattle-based economy has been linked to increased milk consumption based on the recurrent detection of dairy residues associated with early Neolithic pottery^56^, traces of which have recently also been detected in some Swifterbant Culture potsherds from Bazel (research in progress).

## Conclusions

The present study irrefutably proofs the presence of domesticated sheep/goat and most likely also cattle from ca. 4800/4600 cal BC along the NW margins of the agro-pastoral frontier, and hence supports the model viewing the neolithization of NW Europa as a long-term process. In addition the isotope data, although not yet fully conclusive, seems to be in favor of small-scale husbandry from the very beginning. If this is confirmed by future, more in-depth isotope analyses, it demonstrates that farmer-herders had a considerable impact on hunter-gatherer’s subsistence as early as the first half of the 5th millennium cal BC. Clearly before 4800/4600 cal BC contact and interaction with farmer-herders from the LBK was limited to the exchange of “exotic” commodities, such as decorated pottery. This changed markedly with the development of the subsequent Blicquy/Villeneuve-Saint-Germain and Rössen Cultures. Contact intensified resulting in a transfer of knowledge on pottery production, the production of new stone tools (with new functions) and likely also herding. In this sense the first half of the 5th millennium cal BC was a turning point for hunter-gatherers living in the lowlands along the margins of the agro-pastoral frontier, corresponding to the “substitution phase”^5,6^ or “Introduction phase”^17^. It was the start of a totally new lifeway which probably would culminate into a fully agrarian society in the course of the second half of the 5th millennium cal BC, around 4000 cal BC at the latest. The latter might have been triggered by the tidal flooding events in the Lower Scheldt valley which certainly will have reduced the availability of edible plants and wild game considerably. On the other hand flooding might have offered better conditions for local agriculture, through the deposition of more fertile tidal mud, including clay and silt^47^, in an overall sandy environment.

## Materials and methods

### Context information

Animal bones at Bazel “Sluis” have been found in two topographical and stratigraphical positions. The largest quantity of bones was retrieved from a peat and fluvial sand deposit along the former bank of the channel^30^ and can be considered as a dump. The remaining bones were found spread over de top of the sandy elevation intermixed with the other cultural remains. There is a marked difference between both sets of bones, as those of the bank dump are much better preserved and include larger bones, while those from the higher grounds are often reduced to small fragments and teeth^25^.

### Bone identification

Faunal remains were identified using standard zooarchaeological techniques^57–59^ and by using several reference collections. Specimens were identified to species level, where possible, and to body size categories (e.g., large or medium mammal) in the case of less identifiable specimens. Boessneck^60^ was used to distinguish between sheep and goat. Differentiation between cattle and aurochs was initially based on overall dimensions and size and subsequently measurements were compared to references in literature^61–69^. The distinction between pig and wild boar was far more problematic^25^, hence these were excluded from the present study. Regretfully, attempts to obtain genetic information on some domesticated bones failed.

### Collagen extraction

Collagen was extracted from the bones following the Longin method^70^. The chemical pretreatment consists of a demineralization with 2.4MHCl for 20 min. An alkaline washing with 0.25 M NaOH for 15 min is performed to remove contaminants like fulvic and humic acids that can be present in the bone matrix. A final rinse with 0.3 M HCl is performed to remove any calcium carbonate precipitation. Each treatment is followed by a Milli-Q water rinsing using an Ezee-Filter separator, allowing a better conservation of the sample and a faster process. The sample is then soaked in a closed test tube containing a pH = 3 (HCl 10 − 3 M) solution at 90 °C for 10 h to obtain the total extraction of the collagen protein. The remaining solid is separated from the solution with a Büchner funnel by vacuum suction with glass fiber prefilters (7 μm, Merck–Millipore). The solution containing the collagen protein is then freeze-dried for 10–15 h.

### Radiocarbon dating

On top of the four earlier dated samples^25^ an extra 12 bones and teeth from domesticated species were radiocarbon dated (Table 2; Fig. 3). Due to problems in separating bones from pig and wild boar, the selection mainly focused on fragments from cattle (10) and to a lesser extent sheep/goat (2).

Samples were transferred into quartz tubes with CuO and Ag and combusted to CO2. Graphitization of CO2 was carried out usingH2 over a Fe catalyst. Targets were prepared at the Royal Institute for Cultural Heritage in Brussels (Belgium)^71^ and ^14^C concentrations were measured with accelerator mass spectrometry (AMS) at the Leibniz Labor für Altersbestimmung und Isotopenforschung in Kiel (lab-code KIA) (Germany)^72^ or at the Royal Institute for Cultural Heritage, Brussels, Belgium (lab-code RICH)^73^. ^14^C results are expressed in pMC (percentage modern carbon) and indicate the percent of modern (1950) carbon normalized to δ^13^C = –25‰ using the δ^13^C measurements^74^. Calibration and modelling of the dates were performed using the IntCal13 curve^75^ and the Sum-function in the OxCal online version 4.3.

### Stable isotopes (δ^13^C and δ^15^N), %C, %N and C:N ratio

Carbon and nitrogen stable isotope compositions were measured as the ratios of the heavy isotope to the light isotope (^13^C/^12^C or ^15^ N/^14^ N) and are reported in delta (δ) notation as parts per thousand (‰), where δ^13^C or δ^15^N = ([Rsample/Rstandard]—1) × 1000, and R is ^13^C/^12^C or ^15^ N/^14^ N, relative to internationally defined standards for carbon (Vienna Pee Dee Belemnite, VPDB) and nitrogen (Ambient Inhalable Reservoir, AIR). Analyses were performed in duplicate on a Thermo Flash EA/HT elemental analyser, coupled to a Thermo DeltaV Advantage Isotope Ratio Mass Spectrometer via ConfloIV interface (ThermoFisher Scientific, Bremen, Germany). Standards used were IAEA-N1, IAEA-C6, and internally calibrated acetanilide. Analytical precision was 0.25‰ for both δ^13^C and δ^15^N based on multiple measurements of the standard acetanilide.

### Strontium isotope ratios and concentrations of tooth enamel

Prior to analyses, the external layers of enamel were removed using a diamond drill as the external layers of enamel are likely to be contaminated with exogenous strontium^76–79^. About 50 mg of tooth enamel powder was then collected using a diamond drill. The powdered samples were then pre-treated using 0.1 M acetic acid in excess for 30 min, then rinsed three times with MilliQ^80^.

About 15 mg of pre-treated enamel were then used for strontium isotope analyses, placed in a Teflon beaker and digested in 1 mL of subboiled 14 M HNO3. After complete dissolution, the samples were left to dry on a hotplate at 100 °C until dryness. Strontium was extracted from the samples and purified following the protocol described in Snoeck et al.^81^ and measured on a Nu Plasma MC-ICP Mass Spectrometer (Nu015 from Nu Instruments, Wrexham, UK) at the Université Libre de Bruxelles (ULB). During the course of this study, repeated measurements of the NBS987 standard yielded ^87^Sr/^86^Sr = 0.710246 ± 45 (2SD for > 300 analyses), which is, for our purposes, sufficiently consistent with the mean value of 0.710252 ± 13 (2SD for 88 analyses) obtained by TIMS (Thermal Ionization Mass Spectrometry) instrumentation^82^. All the sample measurements were normalised using a standard bracketing method with the recommended value of ^87^Sr/^86^Sr = 0.710248^82^. Procedural blanks were considered negligible (total Sr (V) of max 0.02 versus 7–8 V for analyses; i.e. ≈ 0.3%). For each sample the ^87^Sr/^86^Sr value is reported with a 2σ error (absolute error value of the individual sample analysis – internal error).

Using a fraction of the digested samples (see strontium isotope analyses), Sr and Ca concentrations in the sample digests were determined using a Thermo Scientific Element 2 sector field ICP mass spectrometer at the Vrije Universiteit Brussel (VUB), Belgium, in low (88Sr) and medium (42Ca) resolution using Indium (In) as an internal standard and external calibration versus various reference materials (SRM1400, CCB01). The actual strontium concentrations were then calculated by normalizing the calcium data to 40%. Accuracy was evaluated by the simultaneous analysis of two internal bioapatite standards (ENF and CBA). Based on repeated digestion and measurement of these reference materials, the analytical precision of the procedure outlined above is estimated to be better than 5% (1SD, n = 33 for CBA and n = 5 for ENF).

### Carbon and oxygen isotope ratios of tooth enamel

About 1 mg of pre-treated enamel powder (see above) was placed in glass vial and the carbon and oxygen isotope ratios measured on a Nu Perspective Isotope Ratio Mass Spectrometer (IRMS) with a NuCarb carbonate preparation device at the Vrije Universiteit Brussel (VUB, Brussels, Belgium). Internal standards ENF (modern horse tooth enamel) and CBA (modern caw cremated bone) were used (see de Winter et al. 2016 for more details) as well as Iso-Analytical IA-R022. The analytical precision for both carbon and oxygen isotope ratios is ± 0.25‰ or better based on repeated measurements of CBA.

## Supplementary information

Supplementary Information.

## Data Availability

The datasets generated during and/or analyzed during the current study are available from the corresponding author on reasonable request.
