# Supplementary material for: New evidence on the earliest domesticated animals and possible small-scale husbandry in Atlantic NW Europe
Source: Sci Rep. 2020 Nov 18;10:20083. doi: 10.1038/s41598-020-77002-4 (PMC7676240; doi:10.1038/s41598-020-77002-4)
Supplement: Supplementary file 1 — Supplementary Information. [file 41598_2020_77002_MOESM1_ESM.docx]

**Supplementary information S1**

**New evidence on the earliest domesticated animals and possible small-scale husbandry in Atlantic NW Europe**

**Philippe Crombé***, Department of Archaeology, Ghent University, Sint-Pietersnieuwstraat 35, 9000 Ghent (Belgium) [Philippe.crombe@ugent.be](mailto:Philippe.crombe@ugent.be)

**Kim Aluwé**, Gate bvba, Dorpsstraat 73, B-8450 Bredene (Belgium); Department of Archaeology, Ghent University, Sint-Pietersnieuwstraat 35, 9000 Ghent (Belgium) [kim.aluwe@outlook.be](mailto:kim.aluwe@outlook.be)

**Mathieu Boudin**, Royal Institute for Cultural Heritage, Jubelpark 1, 1000 Brussels (Belgium) [Mathieu.boudin@kikirpa.be](mailto:Mathieu.boudin@kikirpa.be)

**Christophe Snoeck**, Research Unit: Analytical, Environmental & Geo-Chemistry, Dept. of Chemistry, Vrije Universiteit Brussel, AMGC-WE-VUB, Pleinlaan 2, 1050 Brussels, Belgium; G-Time Laboratory, Université Libre de Bruxelles, ULB, CP 160/02, 50, Avenue F.D. Roosevelt, B-1050 Brussels, Belgium; Maritime Cultures Research Institute, Dept. of Art Sciences & Archaeology, Vrije Universiteit Brussel, MARI-LW-VUB, Pleinlaan 2, 1050 Brussels, Belgium [Christophe.Snoeck@vub.be](mailto:Christophe.Snoeck@vub.be)

**Liesbeth Messiaen**, Department of Archaeology, Ghent University, Sint-Pietersnieuwstraat 35, 9000 Ghent (Belgium) [Liesbeth.messiaen@ugent.be](mailto:Liesbeth.messiaen@ugent.be)

**Dimitri Teetaert**, Department of Archaeology, Ghent University, Sint-Pietersnieuwstraat 35, 9000 Ghent (Belgium) [Dimitri.teetaert@ugent.be](mailto:Dimitri.teetaert@ugent.be)

**Detailed species composition**

|  | **NISP(2016)** | **NISP%(2016)** | **NISP(2020)** | **NISP%(2020)** | **NISP** | **NISP%** |
| --- | --- | --- | --- | --- | --- | --- |
| aurochs | 43 | 10,2% | 1 | 0,1% | 44 | 3,1% |
| cattle | 75 | 17,9% | 117 | 11,8% | 192 | 13,6% |
| aurochs/cattle | 39 | 9,3% | 0 | 0,0% | 39 | 2,8% |
| sheep | 2 | 0,5% | 2 | 0,2% | 4 | 0,3% |
| goat | 0 | 0,0% | 1 | 0,1% | 1 | 0,1% |
| sheep/goat | 13 | 3,1% | 5 | 0,5% | 18 | 1,3% |
| wild boar | 1 | 0,2% | 0 | 0,0% | 1 | 0,1% |
| pig/wild boar | 27 | 6,4% | 71 | 7,1% | 98 | 6,9% |
| red deer | 41 | 9,8% | 2 | 0,2% | 43 | 3,0% |
| roe deer | 1 | 0,2% | 0 | 0,0% | 1 | 0,1% |
| dog | 2 | 0,5% | 5 | 0,5% | 7 | 0,5% |
| hare | 1 | 0,2% | 0 | 0,0% | 1 | 0,1% |
| beaver | 1 | 0,2% | 1 | 0,1% | 2 | 0,1% |
| small mammal | 0 | 0,0% | 1 | 0,1% | 1 | 0,1% |
| medium-sized mammal | 5 | 1,2% | 220 | 22,1% | 225 | 15,9% |
| large mammal | 25 | 6,0% | 147 | 14,8% | 172 | 12,2% |
| human | 1 | 0,2% | 1 | 0,1% | 2 | 0,1% |
| bird | 8 | 1,9% | 0 | 0,0% | 8 | 0,6% |
| indet. | 135 | 32,1% | 421 | 42,3% | 556 | 39,3% |
| TOTAL | 420 |  | 995 |  | 1415 |  |
